# Primary mismatch repair deficient IDH-mutant astrocytoma (PMMRDIA) is a distinct type with a poor prognosis

**DOI:** 10.1007/s00401-020-02243-6

**Published:** 2020-11-20

**Authors:** Abigail K. Suwala, Damian Stichel, Daniel Schrimpf, Matthias Kloor, Annika K. Wefers, Annekathrin Reinhardt, Sybren L. N. Maas, Christian P. Kratz, Leonille Schweizer, Martin Hasselblatt, Matija Snuderl, Malak Sameer J. Abedalthagafi, Zied Abdullaev, Camelia M. Monoranu, Markus Bergmann, Arnulf Pekrun, Christian Freyschlag, Eleonora Aronica, Christof M. Kramm, Felix Hinz, Philipp Sievers, Andrey Korshunov, Marcel Kool, Stefan M. Pfister, Dominik Sturm, David T. W. Jones, Wolfgang Wick, Andreas Unterberg, Christian Hartmann, Andrew Dodgshun, Uri Tabori, Pieter Wesseling, Felix Sahm, Andreas von Deimling, David E. Reuss

**Affiliations:** 1 Department of Neuropathology, Institute of Pathology, Heidelberg University Hospital, Heidelberg, Germany; 2 Clinical Cooperation Unit Neuropathology, German Cancer Research Center (DKFZ), German Consortium for Translational Cancer Research (DKTK), Heidelberg, Germany; 3 Department of Applied Tumor Biology, Institute of Pathology, University Hospital Heidelberg, Heidelberg, Germany; 4 Clinical Cooperation Unit Applied Tumor Biology, German Cancer Research Center (DKFZ), Heidelberg, Germany; 5 Molecular Medicine Partnership Unit (MMPU), University Hospital Heidelberg, European Molecular Biology Laboratory, Heidelberg, Germany; 6 Department of Pathology, University Medical Center Utrecht, Utrecht University, Utrecht, The Netherlands; 7 Department of Pediatric Hematology and Oncology, Hannover Medical School, Hannover, Germany; 8 Department of Neuropathology, Berlin Institute of Health, Charité-Universitätsmedizin Berlin, Corporate Member of Freie Universität Berlin, Humboldt-Universität Zu Berlin, Berlin, Germany; 9 German Cancer Consortium (DKTK), Partner Site Berlin, German Cancer Research Center (DKFZ), Heidelberg, Germany; 10 Institute of Neuropathology, University Hospital Münster, Münster, Germany; 11 Division of Neuropathology, NYU Langone Health, New York, USA; 12 Laura and Isaac Perlmutter Cancer Center, NYU Langone Health, New York, USA; 13 Division of Molecular Pathology and Diagnostics, NYU Langone Health, New York, USA; 14 Pathology Department, Brigham and Women’s Hospital, Harvard Medical School, Boston, MA USA; 15 Genomics Research Department, Saudi Human Genome Project, King Fahad Medical City and King Abdulaziz City for Science and Technology, Riyadh, Saudi Arabia; 16 Laboratory of Pathology, National Cancer Institute, National Institutes of Health, Bethesda, MD USA; 17 Institute of Pathology, Julius-Maximilians-University, Würzburg, Germany; 18 Institute of Clinical Neuropathology, Bremen-Mitte Medical Center, Bremen, Germany; 19 Professor Hess Children‘s Hospital, Klinikum Bremen-Mitte, Bremen, Germany; 20 Department of Neurosurgery, Medical University of Innsbruck, Innsbruck, Austria; 21 Department of (Neuro)Pathology, Amsterdam UMC, University of Amsterdam, Amsterdam Neuroscience, Amsterdam, The Netherlands; 22 Division of Pediatric Hematology and Oncology, University Medical Center Goettingen, Goettingen, Germany; 23 Hopp Children’s Cancer Center (KiTZ), Heidelberg, Germany; 24 Division of Pediatric Neurooncology, German Cancer Research Center (DKFZ), German Cancer Consortium (DKTK), Heidelberg, Germany; 25 Princess Máxima Center for Pediatric Oncology, Utrecht, The Netherlands; 26 Department of Pediatric Oncology, Hematology and Immunology, University Hospital Heidelberg, Heidelberg, Germany; 27 Pediatric Glioma Research Group, German Consortium for Translational Cancer Research (DKTK), German Cancer Research Center (DKFZ), Heidelberg, Germany; 28 Clinical Cooperation Unit Neurooncology, German Cancer Research Center (DKFZ), German Consortium for Translational Cancer Research (DKTK), Heidelberg, Germany; 29 Department of Neurology and Neurooncology Program, National Center for Tumor Diseases, Heidelberg University Hospital, Heidelberg, Germany; 30 Department of Neurosurgery, University Hospital Heidelberg, Heidelberg, Germany; 31 Department of Neuropathology, Institute of Pathology, Hannover Medical School, Hannover, Germany; 32 Department of Paediatrics, University of Otago, Christchurch, New Zealand; 33 Children’s Haematology and Oncology Center, Christchurch Hospital, Christchurch, New Zealand; 34 The Arthur and Sonia Labatt Brain Tumour Research Centre, The Hospital for Sick Children, Toronto, Canada; 35 Division of Haematology and Oncology, Department of Pediatrics, The Hospital for Sick Children, University of Toronto, Toronto, Canada; 36 Department of Medical Biophysics, Faculty of Medicine, University of Toronto, Toronto, Canada; 37 Department of Pathology, Amsterdam University Medical Centers/VUmc and Brain Tumor Center Amsterdam, Amsterdam, The Netherlands

**Keywords:** Glioblastoma, Mismatch repair, CMMRD, Lynch, IDH, ATRX, DNA methylation, Subtype, Prognosis

## Abstract

Diffuse IDH-mutant astrocytoma mostly occurs in adults and carries a favorable prognosis compared to IDH-wildtype malignant gliomas. Acquired mismatch repair deficiency is known to occur in recurrent IDH-mutant gliomas as resistance mechanism towards alkylating chemotherapy. In this multi-institutional study, we report a novel epigenetic group of 32 IDH-mutant gliomas with proven or suspected hereditary mismatch repair deficiency. None of the tumors exhibited a combined 1p/19q deletion. These primary mismatch repair-deficient IDH-mutant astrocytomas (PMMRDIA) were histologically high-grade and were mainly found in children, adolescents and young adults (median age 14 years). Mismatch repair deficiency syndromes (Lynch or Constitutional Mismatch Repair Deficiency Syndrom (CMMRD)) were clinically diagnosed and/or germline mutations in DNA mismatch repair genes (*MLH1*, *MSH6*, *MSH2*) were found in all cases, except one case with a family and personal history of colon cancer and another case with MSH6-deficiency available only as recurrent tumor. Loss of at least one of the mismatch repair proteins was detected via immunohistochemistry in all, but one case analyzed. Tumors displayed a hypermutant genotype and microsatellite instability was present in more than half of the sequenced cases. Integrated somatic mutational and chromosomal copy number analyses showed frequent inactivation of *TP53*, *RB1* and activation of RTK/PI3K/AKT pathways. In contrast to the majority of IDH-mutant gliomas, more than 60% of the samples in our cohort presented with an unmethylated *MGMT* promoter. While the rate of immuno-histochemical ATRX loss was reduced, variants of unknown significance were more frequently detected possibly indicating a higher frequency of ATRX inactivation by protein malfunction. Compared to reference cohorts of other IDH-mutant gliomas, primary mismatch repair-deficient IDH-mutant astrocytomas have by far the worst clinical outcome with a median survival of only 15 months irrespective of histological or molecular features. The findings reveal a so far unknown entity of IDH-mutant astrocytoma with high prognostic relevance. Diagnosis can be established by aligning with the characteristic DNA methylation profile, by DNA-sequencing-based proof of mismatch repair deficiency or immunohistochemically demonstrating loss-of-mismatch repair proteins.

**Electronic supplementary material:**

The online version of this article (10.1007/s00401-020-02243-6) contains supplementary material, which is available to authorized users.

## Introduction

The discovery of mutations in isocitrate dehydrogenase 1 (*IDH1*) or isocitrate dehydrogenase 2 (*IDH2*) genes in glial brain tumors critically shaped the understanding of the clinical importance of molecular differences in gliomas [4, 37, 51]. Today IDH-mutation is a defining criterion for specific types of glioma. The WHO classification of CNS tumors from 2016 recognizes the following types: diffuse astrocytoma, IDH-mutant (WHO grade II), anaplastic astrocytoma, IDH-mutant (WHO grade III) and glioblastoma, IDH-mutant (WHO grade IV), oligodendroglioma, IDH-mutant and 1p/19q-codeleted (WHO grade II) and anaplastic oligodendroglioma, IDH-mutant and 1p/19q-codeleted (WHO grade III) [35]. Importantly, all types of IDH-mutant gliomas identified have in common that they have a significantly better outcome compared to malignant diffuse IDH-wildtype (IDH-wt) gliomas like glioblastoma, IDH-wt or IDH-wt/H3-mutant gliomas [32, 48]. By DNA methylation profiling, IDH-mutant gliomas are generally clearly distinguishable from IDH-wt tumors by the CpG island methylator phenotype (G-CIMP) [50]. G-CIMP is considered to develop due to IDH mutation-induced production of 2-hydroxyglutarate and its subsequent effects on DNA methylation [45]. The current version of the DNA methylation-based CNS tumor classification system distinguishes three subclasses within the methylation class family glioma, IDH-mutant: subclass astrocytoma (mostly accounts for WHO grade II and III), subclass high-grade astrocytoma (mostly accounts for WHO grade III and IV) and subclass 1p/19q-codeleted oligodendroglioma (including both WHO grade II and III) [14]. Only recently infratentorial astrocytomas were found to be a discrete subgroup within IDH-mutant astrocytomas that also form a distinct methylation cluster [5].

Standard treatment protocols for patients with malignant gliomas include surgery followed by radiotherapy and chemotherapy [8, 43, 46]. The most commonly used chemotherapeutic, temozolomide (TMZ), is an alkylating agent. The modification with the highest cytotoxicity induced by TMZ is alkylation of the oxygen atom O6 of guanine residues (O6-meG), hence leading to mispairing of guanine with thymine during DNA replication [19]. The O6-meG/T mismatch is recognized by the mismatch repair (MMR) system initiating futile cycles of the cellular MMR machinery leading to DNA single- and double-strand breaks and eventually to cell death [17, 41]. The enzyme O(6)-Methylguanine-DNA methyltransferase (MGMT) detoxifies the O6-meG/T mismatch by removing the methyl groups from the O6 position of guanine thereby limiting the effects of an alkylating drug. Promoter methylation-mediated silencing of *MGMT* is associated with increased sensitivity towards temozolomide and present in the vast majority of IDH-mutant gliomas [19]. A fraction of recurrent IDH-mutant gliomas develops resistance against TMZ by acquiring mutations in MMR genes, leading secondarily to a hypermutated genotype [10, 13, 36, 44, 47].

Germline mutations in the MMR genes lead to tumor syndromes known as Lynch syndrome (monoallelic inactivation) and Constitutional Mismatch Repair Deficiency (CMMRD, biallelic inactivation). Patients with these syndromes harbor a high risk of developing a range of cancers [29]. Comparatively little is known about the precise nature of brain tumors in the setting of MMR-deficiency syndromes where IDH-mutant gliomas have rarely been described. Recently, the European C4CMMRD consortium reported about brain tumors occurring in patients with CMMRD. Notably, the vast majority of these tumors were high-grade gliomas but from 26 tumors available for histological review, only one was identified as IDH-mutant [24]. A recent case report described the concomitant occurrence of an IDH wildtype and an IDH-mutant glioma in a patient with CMMRD [22]. Very recently, 51 germline-driven replication repair-deficient high-grade gliomas were analyzed by DNA methylation profiling and were found to be heterogenous including a subset of 6 tumors with IDH mutations [18].

Here, we present data for 32 tumors with proven or suspected primary MMR deficiency forming a novel epigenetic group of IDH-mutant gliomas with an astrocytic phenotype and distinct molecular and clinical parameters including an aggressive biological behavior.

## Materials and methods

### Tissue samples

Samples were collected from university hospitals in Heidelberg, Utrecht, Toronto, Melbourne, Hannover, Münster, New York City, Berlin, Innsbruck, Edinburgh, Würzburg, Göttingen, Freiburg, Hong Kong, the Institute of Cancer Research UK, the National Cancer Institute in Bethesda, Bremen Mitte hospital, and Helios Krefeld hospital. Cases were selected based on t-SNE analysis of genome-wide DNA methylation data from a cohort of more than 70,000 tumors that revealed group formation based on similarities in DNA methylation profiles. EDTA–blood was used for detection of germline variants (*n* = 6). For the reference, cohort samples with clinical data and a high classifier score (> 0.9) and by DNA methylation-based CNS tumor classification were selected. Tissue and data collection were performed in consideration of local ethics regulations and approval.

### DNA extraction

Tumor DNA was extracted from areas with highest tumor cell content using the automated Maxwell system with the Maxwell 16 Tissue DNA Purification Kit or the Maxwell 16 FFPE Plus LEV DNA Purification Kit (Promega, Madison, USA), according to the manufacturer’s instructions. DNA extraction from EDTA–blood was done using the Maxwell RSC Blood DNA Kit (Promega). DNA concentrations were determined using the Invitrogen Qubit dsDNA BR Assay Kit (Thermo Fisher Scientific, Waltham, USA) on a FLUOstar Omega Microplate Reader (BMG Labtech GmbH, Ortenberg, Germany).

### DNA methylation and t-distributed stochastic neighbour embedding (t-SNE) analysis

DNA methylation profiles were generated using the Infinium HumanMethylation450 (450 k) BeadChip or Infinium MethylationEPIC (850 k) BeadChip array (Illumina, San Diego, USA) according to the manufacturer’s instructions. The data were processed as previously described [14]. T-SNE analysis was performed using the 20,000 most variable CpG sites according to standard deviation, 3000 iterations and a perplexity value of 10.

### MGMT promoter methylation analysis

*MGMT* promoter methylation status was calculated from the 450 k/850 k data as described by Bady et al. [2] with some modifications; an individual confidence interval was determined for each single probe; if the cut-off value of 0.358 was located in the calculated confidence interval, the *MGMT* promoter methylation status was defined as being not determinable. Samples with a not determinable *MGMT* promoter are not included in Fig. 2c.

### Copy number profiling

Copy number profiles were generated from the methylation array data using the ‘conumee’ package in R (http://bioconductor.org/packages/release/bioc/html/conumee.html) with additional baseline correction. Summary copy number plots were generated by condensing multiple copy number plots.

### Gene sequencing and mutational burden

For 76 probes (including reference cases), next-generation sequencing was performed on a NextSeq sequencer 500 (Illumina) as described previously [42]. Libraries were enriched by hybrid capture with custom biotinylated RNA oligo pools covering exonic regions of either 130 or 171 genes, respectively. Oncoprint displays exonic and splicing indels and nonsynonymous single-nucleotide variants (SNVs) for selected genes detected in a tumor sample after subtracting low-quality calls and SNVs with a frequency of ≤ 0.001 in the 1000 genomes database (https://www.internationalgenome.org/). Cases 10 and 11 were sequenced using the Ion AmpliSeq™ Cancer Hotspot Panel v2. Case 14 was sequenced with an Ion Torrent NGS custom amplicon panel targeting 56 genes (both Thermo Fisher Scientific). For case 15, exome sequencing was performed. Mutational burden was calculated for samples where sequencing covered more than 0.9 Mb. It indicates the relative number of called exonic and splicing SNVs and indels in a sample per covered megabase. Low-quality calls and SNVs with a frequency of ≤ 0.001 in the 1000 genomes database were excluded. IDH-sequencing was performed as previously described [25].

### Immunohistochemistry

For cases with sufficient material (*n* = 11), immunohistochemistry was conducted on 3 µm thick FFPE tissue sections mounted on StarFrost Advanced Adhesive slides (Engelbrecht, Kassel, Germany) followed by drying at 80 °C for 15 min. Immunohistochemistry was performed on a BenchMark Ultra immunostainer (Ventana Medical Systems, Tucson, USA). Slides were pretreated with Cell Conditioning Solution CC1 (Ventana Medical Systems) for 32 min at room temperature. Primary antibodies were incubated at 37 °C for 32 min, followed by Ventana standard signal amplification, UltraWash, counter-staining with one drop of hematoxylin for 4 min, and one drop of bluing reagent for 4 min. UltraView Universal DAB Detection Kit (Ventana Medical Systems) was used for visualization. Primary antibodies were diluted as followed: MutL1 (MLH1, 1:100, DAKO (Agilent), Santa Clara, USA), MutS2 (MSH2, 1:50, DAKO), MutS6 (MSH6, 1:500, DAKO), PMS2 (1:400, DAKO), ATRX (1:2000, BSB3296, BioSB, Santa Barbara, USA), IDH1 R132H (1:2, clone 1 [15]), Olig2 (1:100, Abcam, Cambridge, UK), GFAP (1:2000, Cell signalling, Promega). Stained slides were scanned on the Aperio AT2 Scanner (Aperio Technologies, Vista, USA) and digitalized using Aperio ImageScope software v12.3.2.8013.

### Microsatellite analysis

For MSI analysis, polymerase chain reaction with fluorescently labelled oligonucleotides was performed to amplify the mononucleotide markers BAT25, BAT26, and CAT25 from tumor tissue DNA, as described previously [20]. Amplified fragments were visualized on an ABI3130*xl* genetic analyzer (Thermo Fisher Scientific) to detect potential length alterations of the microsatellites. Fragment sizes differing from the normal range of allelic size variation known for the amplicons encompassing BAT25 (108–110 bp), BAT26 (116–118 bp), and CAT25 (146–148 bp) were regarded as microsatellite instability if no normal tissue DNA was available as a reference. Tumors showing microsatellite instability in one or more markers were classified as microsatellite-instable.

### Statistical analysis

Sample sizes (*n*) and statistical tests are indicated in figure legends. Kaplan–Meier curves were created and log-rank tests calculated using Prism 8 (GraphPad Software, La Jolla, USA). Overall survival was defined as the time between first surgery and death or the last follow-up for all cases except case 17. For case 17 survival was calculated starting from diagnosis of the recurrent tumor as this was the only material available for analyses. For the Kaplan–Meier curve comparing the overall survival of patients with PMMRDIA and IDH-wt high-grade gliomas in CMMRD data from Guerrini-Rousseau et al., 2019 [24] were extracted. Data from patients with anaplastic astrocytoma or glioblastoma were included, excluding IDH-mutant tumors.

## Results

### DNA methylation profiling reveals a distinct group of IDH-mutant gliomas

Using unsupervised t-distributed stochastic neighbor embedding (t-SNE) analysis of DNA methylation profiles of an extensive set (> 70,000) of CNS and non-CNS tumors, we identified a distinct group of 32 samples clustering closely together, neighboring but not overlapping with IDH-mutant astrocytomas (data not shown). Results of DNA methylation-based CNS tumor classification (version11b4) did not show any matching calibrated scores except for one tumor classified as “methylation class IDH glioma, subclass high-grade astrocytoma”. The histological diagnoses for tumors of this group consisted predominantly of WHO grade III and IV astrocytic gliomas (mainly glioblastoma WHO grade IV). The histological diagnoses of anaplastic oligodendroglioma WHO grade III, primitive neuroectodermal tumor (PNET) WHO grade IV and anaplastic ependymoma WHO grade III were given once each before the WHO 2016 classification update was released. This suggested that the histological appearance is heterogenous and that while an astrocytic phenotype predominates in most cases, the lineage is less obvious in some. In most cases, an IDH-mutation of the tumor was documented in the clinical records.

**Fig. 1:**
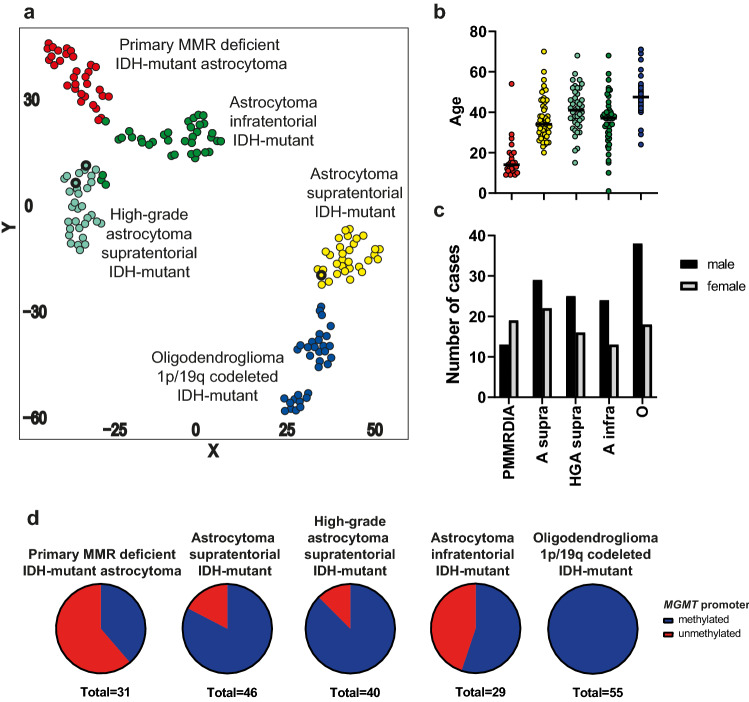
Primary mismatch repair-deficient IDH-mutant astrocytomas form a distinct methylation group. **a** t-distributed stochastic neighbour embedding (t-SNE) analysis of 32 primary MMR-deficient IDH-mutant astrocytomas with 128 reference cases from 4 methylation groups of IDH-mutant gliomas (*n* = 32 for each methylation group). Recurrent hypermutant tumors with treatment induced MMR defects in reference cohort are marked with a bold black border. **b** Age distribution of primary MMR-deficient astrocytoma cases (*n* = 19) compared to reference cohort. Black line represents median (PMMRDIA, 14y; A supra, 34y; HGA supra, 41y; A infra, 37y; O, 47,5). Age of patients with PMMRDIA is significantly younger compared to all other groups (*p* < 0.0001). **c** Gender ratio of primary MMR-deficient astrocytoma cases (*n* = 32) compared to reference cohort (A supra *n* = 51; HGA supra *n* = 41; A infra *n* = 37; O *n* = 56). **d** *MGMT* promoter methylation status assessed by methylation profiling. *PMMRDIA* primary MMR-deficient astrocytoma, *A supra* astrocytoma supratentorial, *HGA supra* high-grade astrocytoma supratentorial, *A infra* astrocytoma infratentorial, *O* oligodendroglioma 1p/19q codeleted

When restricting t-SNE analysis to IDH-mutant gliomas, this newly identified group clearly separated from all other previously described types and subtypes of IDH-mutant glioma (Fig. 1a). However, similar to conventional high-grade supratentorial IDH-mutant astrocytomas, this group displayed a reduced global hypermethylation compared to low-grade supratentorial astrocytomas and oligodendrogliomas (Supplementary Fig. 1, online resource).

### Tumors commonly occur in adolescence

To outline the clinico-pathological characteristics of this epigenetic group, a reference cohort of 185 IDH-mutant tumors representing all previously described epigenetic groups of IDH-mutant glioma was used. The age of patients with tumors of this group was significantly younger with a median age of 14 years (*p* < 0.0001; Fig. 1b). In a prospective registry of newly diagnosed pediatric CNS tumor patients (age 21 and younger), 33% (5/15) of patients diagnosed with IDH-mutant high-grade gliomas were part of this newly identified group (data not shown). In contrast to all other types of IDH-mutant glioma, there were more female than male patients in this group (Fig. 1c). All but one of the tumors were located supratentorial (Table 1).

**Table 1:** Patient characteristics of primary mismatch repair-deficient IDH-mutant astrocytomas

No	Sex	Age	Mut.	Syndrome	P/R	MSI	Mut/Mb	OS	PFS	Death	MGMT	Loc.	Diagn.	Grade	IDH1	ATRX	Additional tumors
1	f	20	MSH6	cMMRD	P	MSI	26	10	10	No	meth	H	EPN	III	R132H	−	CRC with MSI, NHL
2	f	27	MLH1		P	MSI	23	27	3	Yes	not det	H	astro	III	R132H	+	IDH wt GBM
3	f	15	MSH6	cMMRD	P		23	1	1	Yes	meth	H	GBM	IV	R132H	−	
4	f	29	MSH6		P	MSS	16	15	10	Yes	meth	H	oligo	III	R132C	+	
5	f	15	MSH2		U	MSI	23			Yes	unmeth	H	PNET	IV	R132H		
6	f	9	MSH6	Lynch	P	MSI	16	12	8	Yes	unmeth	H	GBM	IV	R132H	−	
7	f	11	MSH6	cMMRD	P	MSI	16	12	11	Yes	meth	H	GBM	IV	R132H	−	
8	f	9	MSH6	cMMRD	P	MSS	8				unmeth	H	GBM	IV	R132H		CRC
9	f		MSH6		U	MSS	5				meth		HGG		R132H		
10	m	54			P			16	10	Yes	unmeth		GBM	IV	R132C	+	CRC
11	m	19		cMMRD	P			16	10	Yes	meth	H	GBM	IV			
12	m		MSH6		U	MSS	12				unmeth		HGG		R132H		
13	m	12	MSH6	cMMRD	P		8	13	13	No	unmeth	H	GBM	IV	R132H	−	
14	m	17	MSH2	Lynch	P			12	6	Yes	unmeth	H	astro	II	R132H		
15	m	14	MSH2	Lynch	P		9	6		No	unmeth	H	GBM	IV	R132H		
16	f	10	MSH6		P	MSI	14	16	11	Yes	unmeth	H	astro	III	R132H	+	
17	m	24			R		4	27	27	No	meth	H	GBM	IV	R132H	−	
18	m	20			P			30	10	Yes	unmeth	H	astro	III	R132H	−	
19	m	13	MSH6	cMMRD	P		9	10		Yes	unmeth	H	astro	III			
20	f	12	MLH1	Lynch	P		8	10		No	meth	H	astro	III			
21	f	12	MSH6	cMMRD	P		11	13		Yes	unmeth	PF	GBM	IV			
22	f	15	MSH6	Lynch	P			2	2	No	meth	H	GBM	IV			
23	f	9			P			10		Yes	unmeth	H	astro	III	R132H	-	
24	m	13			P					Yes	unmeth	H	GBM	IV	R132H		
25	f	11			P			9	9	Yes	unmeth		GBM	IV	R132H	-	
26	f				U						unmeth						
27	f				U						meth						
28	m				U						unmeth						
29	f				U						meth						
30	m				U						meth						
31	f				U						unmeth						
32	m				U						unmeth						

*No.* case number, *Mut.* mutation (MMR defect detected via panel sequencing or immunohistochemistry), *P/R* primary/recurrence, *U* unknown, *MSI* microsatellite instability/instable, *MSS* microsatellite stable, *OS* overall survival [in months], *PFS* progression free survival [in months], *meth* methylated, *unmeth* unmethylated, *not det* not determinable, *Loc* location, *H* hemispheric, *PF* posterior fossa, *Diagn.* histological diagnosis, *EPN* ependymoma, *astro* astrocytoma, *oligo* oligodendroglioma, *GBM* glioblastoma, *HGG* high-grade glioma, *ATRX* ATRX expression as detected by immunohistochemistry, *CRC* colorectal cancer, *NHL* Non-Hodgkin lymphoma

### Tumors display a high proportion of unmethylated MGMT promoter

Hypermethylation of the MGMT promoter is in general a typical feature of IDH-mutant gliomas, except the recently described subtype of infratentorial IDH-mutant astrocytomas [5]. Tumors of the newly identified group demonstrate the highest frequency of an unmethylated MGMT promoter among all IDH-mutant gliomas (61.3%, 19/31, Fig. 1d).

### Histology shows broad range of anaplastic features and a reduced frequency of ATRX loss

**Fig. 2:**
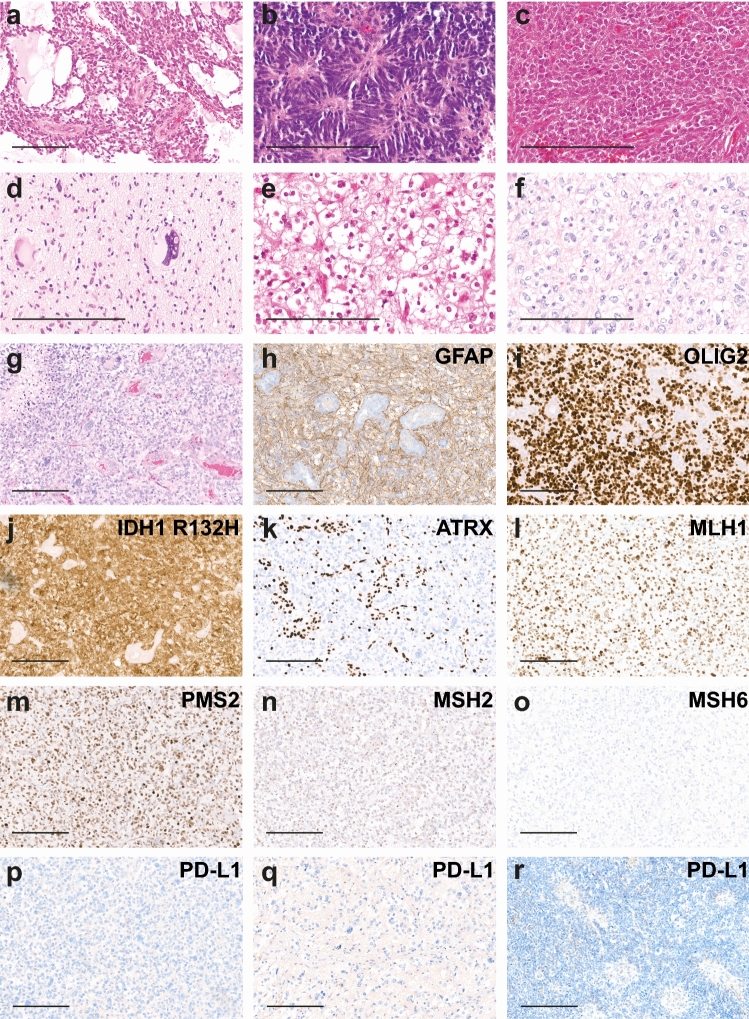
Histology and immunohistology of primary mismatch repair-deficient IDH-mutant astrocytoma. Most tumors have a poorly differentiated astrocytic appearance with little cytoplasm and sparse cellular processes (**a**–**g**). Pseudocysts in case no. 2 (**a**), pseudorosettes in case no. 1, histologically diagnosed as ependymoma (**b**), poor differentiation in case no. 6 (**c**), multinucleated giants cells in case no. 16 (**d**) and oligoid features in case no. 3 and 7 (**e**,** f**) were observed. Endothelial proliferation and necrosis as in case no. 7 (**g**) were present in most cases. Immunohistochemical findings of case no. 7. The tumor shows positivity for GFAP (**h**), Olig2 (**i**), IDH1 R132H (**j**), loss of ATRX expression in tumor cell nuclei (**k**), expression of MLH1 (**l**), PMS2 (**m**), faint expression of MSH2 (**n**) and no expression of MSH6 (**o**) in all cells. No PD-L1 expression of tumor cells in case x no. 7 (**p**), 3 (**q**) and 1 (**r**). Scale bar 200 µm

Histologically, most tumors had a poorly differentiated astrocytic appearance with little cytoplasm and sparse cellular processes reminiscent of small cell astrocytoma or glioblastoma (Fig. 2a). A single tumor exhibited perivascular pseudorosette-like arrangements of tumor cells reminiscent of ependymoma (Fig. 2b). Another tumor presented with an undifferentiated PNET-like appearance (Fig. 2c). Bizarre multinucleated giant cells (Fig. 2d) or oligoid features with perinuclear halos (Fig. 2e, f) were encountered in some tumors. Brisk mitotic activity, endothelial proliferation and necrosis were present in the majority of cases (Fig. 2g, h). Immunohistochemical loss of ATRX expression (Fig. 2k, 69.2%, 9/13) did not appear as frequent as in astrocytoma supratentorial (92%, 46/50, *p* = 0.05, Fisher’s exact test) and high-grade astrocytoma supratentorial (86.8%, 33/38, *p* = 0.154, Fisher’s exact test). However, the differences did not reach statistical significance. In all tumors, GFAP and OLIG2 positivity was present (Fig. 2h, i). Tumors showed no or scarce expression of PD-L1 in a small percentage of tumor cells (Fig. 2p–r). In summary, histology shows a broad morphological range without specific features allowing discrimination from other types of high-grade glioma.

### Primary mismatch repair deficiency and hypermutation is a characteristic feature of the group

**Fig. 3:**
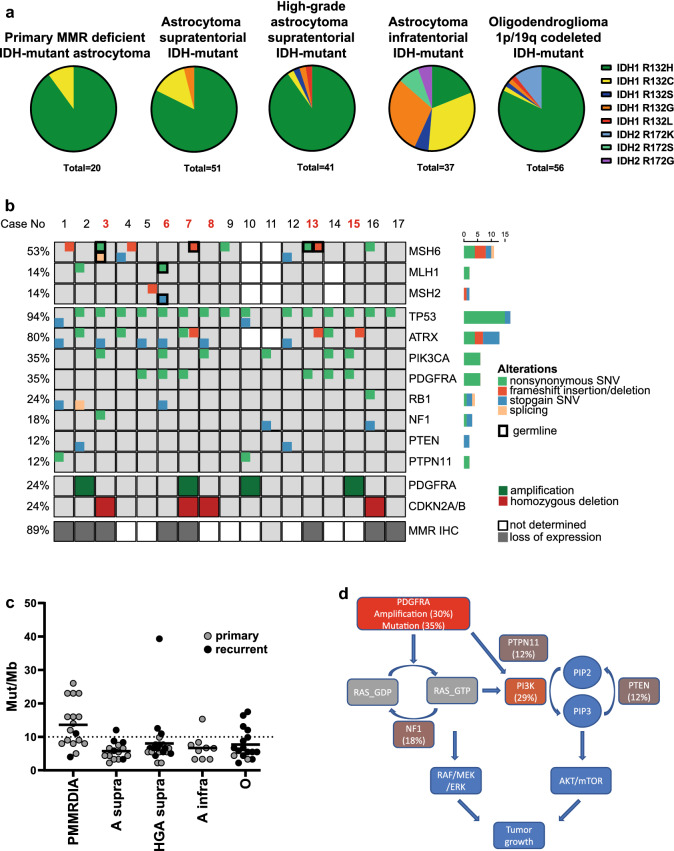
Molecular alterations of primary mismatch repair-deficient IDH-mutant astrocytomas. **a** Distribution of IDH mutations in PMMRDIA compared to reference cohorts. **b** Oncoprint showing selected genetic alterations in PMMRDIA. Red numbers indicate that a blood control was available. **c** Mutational burden in IDH-mutant gliomas (PMMRDIA, primary MMR-deficient IDH-mutant astrocytoma *n* = 17; A supra, astrocytoma supratentorial *n* = 15; HGA supra, high-grade astrocytoma supratentorial *n* = 22; A infra, astrocytoma infratentorial *n* = 9; O, oligodendroglioma 1p/19q codeleted *n* = 17). **d** Pathogenic alterations in PMMRDIA in RAS/PI3K/AKT signaling pathway

To get a deeper insight into the molecular characteristics of this group, we analyzed next-generation sequencing results from 17 cases. Seven cases were analyzed using the Heidelberg 130 gene panel, six cases were sequenced using the Heidelberg 171 gene panel, two cases were analyzed with a 50 gene panel, one case was analyzed using a 56 gene panel and for another case, whole exome sequencing data were available. All cases harbored *IDH1* mutations, 90% of which were IDH1-R132H similar to conventional supratentorial IDH-mutant astrocytomas (Fig. 3a). Inactivating stop-gain or frameshift mutations in one of the MMR genes MutL homolog 1 (*MLH1*), MutS homolog 2 (*MSH2*), and MutS homolog 6 (*MSH6*) were detected in 11/17 tumors (Fig. 3b), and where available (*n* = 6), these mutations could all be identified as germline in origin. For cases 10, 11 and 14 MMR genes were not or only partially covered by the panel sequencing. However, clinical records of case no. 10 describe a personal and a family history of colorectal cancer. Also, case no. 14 and 15 were diagnosed with Lynch syndrome with a known deleterious germline *MSH2* mutation and in case no. 8 and 11 CMMRD was known. For case number 17, paraffin blocks were available, and the tumor showed a tumor cell specific loss of MSH6 expression. This was the only tumor of the series which was available at recurrence only, precluding formal proving of primary MMRD. However, clinical documentation of a primary lesion at young age, immuno-histochemical MSH6 deficiency of the tumor cells and a DNA methylation profile indistinguishable from other samples of this group are still compatible with a Lynch-associated case. Of note, detection of mutations in *PMS2* by panel-sequencing from FFPE derived DNA is hampered by multiple pseudogenes reducing sensitivity [27]. Taken together, these data suggested that likely all tumors of this group occurred in association to an MMR-deficiency syndrome. Indeed, all but one tumor showed loss of expression of at least one MMR protein (88.8%, 8/9, Fig. 2i–o). The single tumor with retained expression of MMR proteins occurred in a child with known CMMRD, likely due to a missense mutation with retained protein expression (case no. 11).

Considering the cut-off for hypermutation at 10 or more somatic mutations per Mb [12], 58.8% (10/17) of primary MMR-deficient IDH-mutant astrocytomas were hypermutant (Fig. 3c). Microsatellite instability is frequently observed in other MMR-deficient solid tumors [38]. More than half of the primary mismatch repair-deficient IDH-mutant astrocytomas tested were microsatellite instable, which was detected in hypermutant tumors only (Table 1).

### DNA methylation profiles of primary and secondary MMR-deficient IDH-mutant gliomas are distinct

Variants in MMR genes in the supratentorial IDH-mutant reference cohort were only detected in pretreated, recurrent tumors except for one case, where the *MSH6* variant was found in a primary supratentorial astrocytoma. However, the pathogenic relevance is unclear since this variant was not found in ClinVar or COSMIC and the tumor was not hypermutant (Table 2). If available, we panel-sequenced the respective primary tumors of the recurrent MMR-mutant tumors and found these to be MMR-wildtype and non-hypermutant consistent with a secondary, therapy-associated MMR-deficient hypermutant status. Importantly, these secondary MMR-deficient IDH-mutant gliomas do not display an aberrant DNA methylation profile compared to MMR-proficient conventional IDH-mutant astrocytomas. In restricted t-SNE analyses, primary and secondary MMR-deficient IDH-mutant astrocytomas were completely separated (Fig. 1a). We concluded that epigenetic profiles associate with MMR-deficiency syndromes but not with MMR deficiency per se. Considering these findings, we suggest to designate this novel group as “Primary Mismatch Repair Deficient IDH-mutant Astrocytoma” (PMMRDIA).

**Table 2:** Distribution of selected variants in IDH-mutant gliomas

Cohort	Primary MMR-deficient astrocytoma	Astrocytoma supratentorial	High grade astrocytoma supratentorial	Astrocytoma infratentorial	Oligodendroglioma 1p/19q codeleted
*n*	17	15	22	9	17
MSH6	53.3% (8/15)	13.3% (2)	9.1% (2)	0	0
MLH1	14.3% (2/14)	0	0	0	5.9% (1)
MSH2	14.3% (2/14)	0	0	0	0
TP53	94.1% (16/17)	80% (12)	95.5% (21)	88.9% (8)	5.9% (1)
ATRX	80% (12/15)	53.3% (8)	59.1 (13)	33.3% (3)	0
PDGFRA	35.3% (6/17)	0	9.1% (3)	11.1% (1)	5.9% (1)
PIK3CA	35.3% (6/17)	0	18.2% (4)	11.1% (1)	5.9% (1)
RB1	23.5% (4/17)	13.3% (2)	4.6% (1)	0	0
NF1	17.6% (3/17)	0	9.1% (3)	11.1% (1)	0
PTEN	11.8% (2/17)	0	0	0	0
PTPN11	11.8% (2/17)	0	0	0	0

### Integrated mutational and chromosomal copy number analyses show frequent inactivation of TP53, RB1 and activation of RTK/PI3K/AKT pathways

**Fig. 4:**
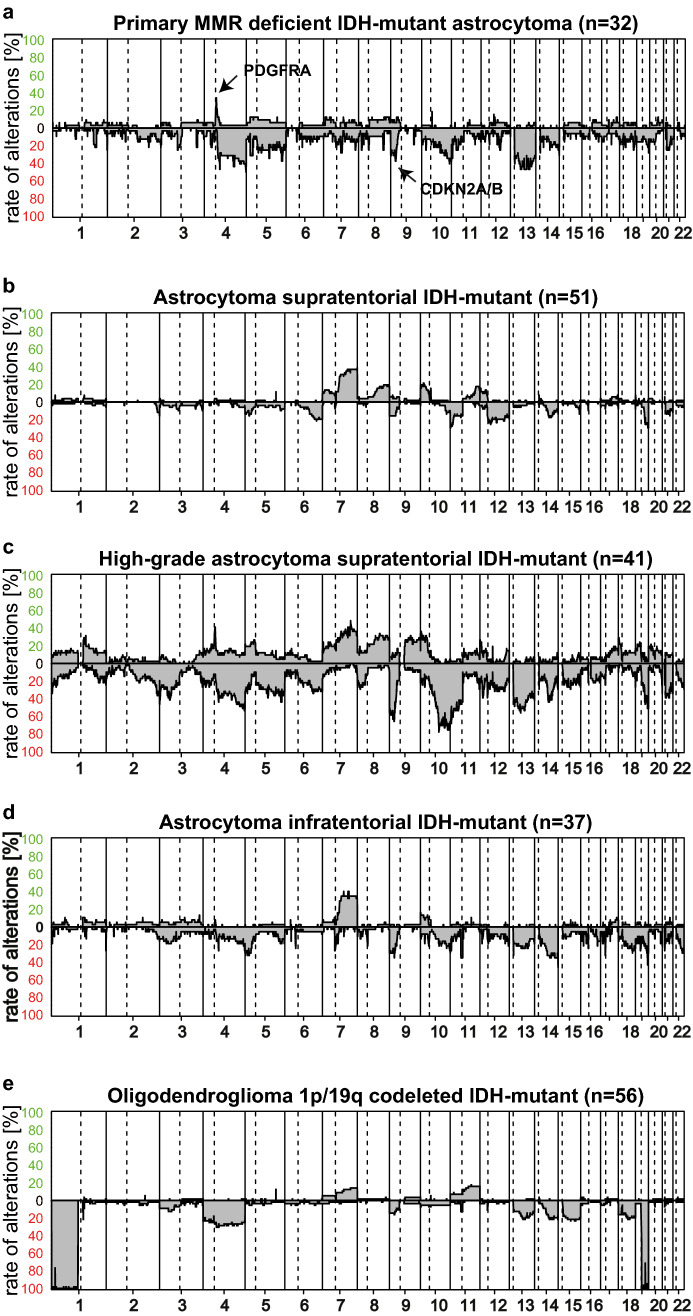
Summary copy number plots of primary mismatch repair-deficient IDH-mutant astrocytomas compared to IDH-mutant reference classes. **a** PMMRDIA present with frequent *PDGFRA* amplification, loss of chromosome 4q and 13q and more copy number alterations than astrocytomas supratententorial (**b**), but are distinct compared to high-grade astrocytomas supratentorial (**c**), astrocytomas infratentorial (**d**) and oligodendrogliomas 1p/19q codeleted (**e**)

Next, we analyzed which genes are recurrently altered in PMMRDIA beside *IDH1* and MMR genes by point mutations or small insertions/deletions and which chromosomal copy number alterations are present. Somatic mutations in *TP53* were present in all but one PMMRDIA (Fig. 3b). Bi-allelic inactivation of *TP53* was evident in the majority of cases, either by the presence of two pathogenic missense mutations or the combination of a pathogenic point mutation and monosomy of chromosome 17p (58.8%, 10/17). *ATRX* variants occurred in the vast majority of cases (80%, 12/15) most of which were clearly pathogenic. Of note, several cases showed *ATRX* missense variants of unknown significance (20%, 3/15). Considering a possibly pathogenic relevance of the variants, this may point to a higher frequency of ATRX functional inactivation than estimated by immuno-histochemical loss. Consistent with an astrocytic genomic profile, no promoter *TERT* mutation was detected and no 1p/19q co-deletion was present. While inactivation of *TP53* is typical for the complete spectrum of IDH-mutant astrocytomas, alterations of the retinoblastoma tumor-suppressor gene (*RB1*) or related pathway components have been implicated in the malignant progression of IDH-mutant astrocytomas. Among the different RB1-pathway alterations recognized, the homozygous deletion of 9p including the *CDKN2A/B* locus has the strongest association with poor overall survival in conventional IDH-mutant supratentorial astrocytomas [7]. In PMMRDIA, the *RB1* gene was affected by point mutations more frequently (23.5% vs. 4.6%) whereas deletions of *CDKN2A/B* were present less frequently (35% vs. 70%) compared to the reference cohort of high-grade supratentorial IDH-mutant astrocytomas (Fig. 4).

Activating point mutations in platelet-derived growth factor receptor alpha (*PDGFRA*) were present in 6/17 (35.3%) of PMMRDIA, much more often than in supratentorial, high-grade IDH-mutant astrocytomas (9.1%). Overall, there were fewer chromosomal copy number alterations in PMMRDIA compared to supratentorial high-grade IDH-mutant astrocytomas (4.5 × 10^8^ vs 8.7 × 10^8^, *p* < 0.0001, unpaired t-test). However, segmental amplifications of 4q including *PDGFRA*, often associated with a concomitant loss of larger parts of 4q, were observed with a similar frequency in PMMRDIA (30%) and conventional supratentorial IDH-mutant high-grade astrocytomas (35%, Fig. 4). Of note, *PDGFRA* amplifications and/or activating point mutations were present in about half of all PMMRDIA. Besides one case where a variant of unknown significance was detected, activating mutations in the phosphatidylinositol-4,5-bisphosphate 3-kinase catalytic subunit alpha (*PIK3CA*) were found in 5/17 (29.4%) of PMMRDIA, a higher rate as in conventional supratentorial IDH-mutant high-grade astrocytomas (13,6%, 3/22, Table 2). Additional recurrently mutated genes were *NF1* (3/17), *PTPN11* (2/17) and *PTEN* (2/17) all of which are coding for important regulators of RAS/PI3K/AKT signaling ([34, 40, 52], Fig. 3d). Segmental amplifications of oncogenes included *CDK4* (2/17), *CDK6* (2/17), *MDM4* (1/17), *CCND2* (1/17), *MET* (1/17) and *EGFR* (1/17). Taken together, these data suggest that the vast majority of PMMRDIA harbor genomic alterations leading to increased oncogenic RAS/PI3K/AKT signaling in addition to a combined inactivation of TP53 and RB1 tumor-suppressor pathways.

### Poor overall survival of patients with PMMRDIA

**Fig. 5:**
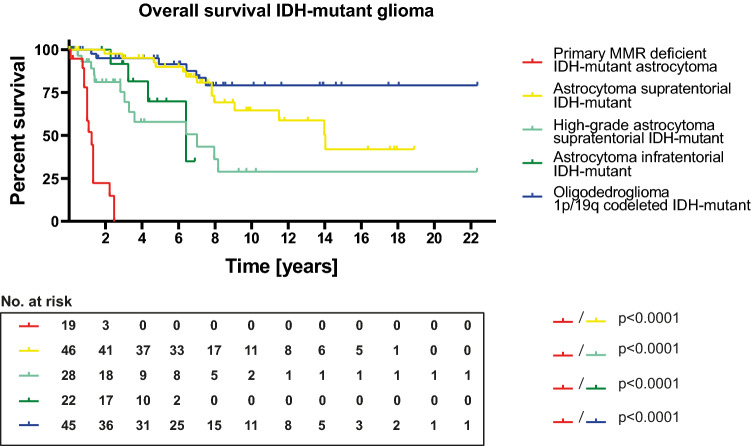
Primary mismatch repair-deficient IDH-mutant astrocytomas have a poor clinical outcome compared to all other IDH-mutant gliomas

Given the well-known favorable outcome of diffuse IDH-mutant gliomas compared to malignant diffuse IDH-wt gliomas, on the one hand, and the known temozolomide-resistant phenotype of MMR-deficient cells, on the other hand, we addressed the question whether the clinical outcome of PMMRDIA significantly differs from that of other IDH-mutant gliomas. Overall survival (OS) data were available for 19 patients. When comparing Kaplan–Meier survival curves, it was striking that PMMRDIA exhibit by far the worse clinical outcome among all IDH-mutant gliomas (Fig. 5). Mean overall survival was only 15 months, compared to 168.4 months for astrocytoma supratentorial (*p* < 0.0001), 85.2 months for high-grade astrocytoma supratentorial (*p* < 0.0001), 76.9 months for astrocytoma infratentorial (*p* < 0.0001) and not defined for oligodendroglioma (*p* < 0.0001, Fig. 5) which is known to have the best prognosis among diffuse gliomas. Compared to data from the literature, this very poor OS is well in the range of adult-type glioblastoma, IDH-wildtype [1] and clearly worse than that for glioblastoma, IDH-mutant [31]. There is no difference between overall survival of PMMRDIA patients compared to CMMRD patients with IDH-wildtype high-grade gliomas published by the European C4CMMRD consortium (Supplementary Fig. 2, online resource, [24]). All except one of the 19 patients with PMMRDIA died within 26 months, including the single patient of the cohort with a WHO grade II tumor which had an OS of only 12 months. Most patients were treated with radio-chemotherapy including TMZ or CCNU (Supplementary Table 1, online resource). Patient 7, 13 and 14 that were diagnosed with an MMR-deficiency syndrome were treated with immune checkpoint inhibitors with limited effect. These patients did not have any further known cancer manifestations. Importantly, patients in our cohort presenting with multiple different tumors due to germline MMR defects (e.g. colon carcinoma or non-Hodgkin-lymphoma) deceased caused by complications of their brain tumor. Given the role of *MGMT* promoter methylation as predictive biomarker for TMZ response in glioblastoma IDH-wt, we compared the patient’s outcome for tumors with and without *MGMT* promoter methylation but did not find a difference (Supplementary Fig. 3a, online resource). A WHO grade IV associates with a significantly worse survival in conventional IDH-mutant astrocytomas. However, Kaplan–Meier analyses of PMMRDIA of WHO grade IV versus WHO grade II/III tumors did not show a significant difference in OS (Supplementary Fig. 3b, online resource). There was, however, an association between homozygous deletion of *CDKN2A/B* and shorter OS in PMMRDIA (Supplementary Fig. 3c, online resource).

## Discussion

Our study reveals that IDH-mutant astrocytomas occurring in children and young adults with germline mutations in MMR genes (Lynch and CMMRD) constitute a distinct entity which should be separated from other IDH-mutant gliomas. This claim is based on several critical differences with important clinical implications. Even though most patients of our cohort received standard combined radio-chemotherapy the outcome was poor, similar to patients with IDH-wt glioblastomas. This suggests that the standard treatment is ineffective which could be attributed at least in part to the presumable primary resistance towards alkylating drugs like TMZ mediated by MMR deficiency. Another adverse feature of PMMRDIA might be the high rate of *MGMT* promoter unmethylated tumors even though no difference in OS depending on the *MGMT* promoter status was visible in our cohort. This may argue for giving patients with PMMRDIA access to first line experimental treatments as increasingly done in patients with *MGMT* unmethylated glioblastoma, IDH-wt [49].

Poly (ADP-ribose) polymerase inhibitors (PARPi) are under investigation as TMZ-sensitizers for the treatment of malignant gliomas in general (e.g. NCT02152982). Interestingly, recent preclinical data showed restoration of TMZ sensitivity specifically in MSH6-deficient glioma cells using PARPi [26]. Even though the mechanism of this PARP1-independent effect remains to be determined, combining TMZ with PARPi could represent an option for the treatment of PMMRDIA.

Another therapeutic approach for MMR-deficient, hypermutant tumors in general is the use of immune checkpoint inhibitors to activate the immune system as they present with an increased number of neoantigens that could be detected by host immune cells [3, 16, 30, 33, 38]. Indeed, case reports of successful treatments of malignant IDH-wt brain tumors in CMMRD with checkpoint inhibitors lead to current clinical studies evaluating the potential benefit of this strategy [6, 28]. Of note, three patients of our cohort received an immune checkpoint inhibitor during the course of disease without notable response. Even though more data are needed for reliable conclusions, this may suggest that immune checkpoint inhibition has limited efficacy in PMMRDIA. Additionally, while most PMMRDIA are indeed hypermutant, the mutational burden is not as high as in tumors usually detected in patients with CMMRD or Lynch syndrome [11]. Another potential barrier for immune-mediated therapies of PMMRDIA could be the fact that the IDH-mutation-associated oncometabolite 2-hydroxyglutarate (2-HG) was shown to strongly repress T-cell activity, possibly impeding containment of the tumor by the immune system [9]. Therefore, IDH-mutation specific inhibitors shown to suppress 2-HG levels could be of interest [39].

Yet another approach could rely on targeted therapies using small molecule inhibitors of activated oncogenes. In this respect, the accumulation of driving alterations in PMMRDIA along the RTK/PI3K/AKT pathway, most commonly affecting *PDGFRA* and *PIK3CA* could represent a starting point for further studies. Regarding the particularly common *PDGFRA* alterations in PMMRDIA, it would be interesting to further evaluate whether their selection is paved by the constitutive enhancer interacting with the PDGFRA gene known to be formed by DNA hypermethylation in IDH-mutant tumors [21].

In addition, a notable fraction of PMMRDIA exhibits inactivation of *RB1*, for which a synthetic lethal interaction with inhibition of aurora A kinase has been suggested [23].

Correct diagnosis of PMMRDIA delineated from other IDH-mutant gliomas is not only important for the prognosis and possible treatment strategy of the affected individual itself, but also for family members due to the tight association of this tumor type with germline MMR deficiency.

Despite the substantial influence of IDH-mutations on the DNA methylation pattern, PMMRDIA were clearly distinguished by DNA methylation profiling from other IDH-mutant gliomas including secondary MMR-deficient tumors which argues for a distinctive cell of origin or an early divergence during oncogenesis. This result differs from those of a recent study using DNA-methylation profiling, in which 6 IDH-mutant gliomas with germline mismatch repair deficiency clustered together with sporadic non-mismatch repair-deficient IDH-mutant astrocytomas [18]. This however, is most likely explainable by the different sizes of the cohorts. The larger cohort of MMR-deficient IDH-mutant astrocytoma in the present study likely allowed the detection of more subtle differences in the DNA methylation profile, which are otherwise obscured in comparison to non-IDH mutant cohorts. Distinctiveness of PMMRDIA profiles is further underscored by the fact that the brain tumor classifier virtually never finds matching scores for these tumors. Upcoming versions of the DNA methylation-based CNS tumor classification will include this methylation class facilitating identification of respective cases.

Beside DNA methylation profiling, the clinical history of another tumor (e.g. colorectal carcinoma) and the age of the patient could hint towards PMMRDIA. Since IDH1-R132H is by far the most frequent IDH-mutation in PMMRDIA, IDH1-R132H-specific antibody is a sensitive tool for detecting PMMRDIA. However, loss of ATRX appears to be less sensitive in PMMRDIA than for conventional supratentorial IDH-mutant astrocytoma to identify cases with a rare IDH-mutation.

PMMRDIA should be considered as a differential diagnosis in all cases of an IDH-mutant tumor with intact 1p/19q or loss of ATRX as a surrogate in a child, adolescent or young adult especially if histology shows high-grade features. In this situation and on condition that the tumor is treatment-naïve, immunohistochemistry for MMR proteins is very helpful. Inclusion of positive controls is important for cases of CMMRD in which all cells present with a protein loss. Loss of expression of at least one MMR protein confirms the diagnosis. Further molecular characterization of the tumor and genetic counseling is then recommended.

With cIMPACT-NOW update 5, a new terminology and a novel grading system were proposed for IDH-mutant astrocytomas. The term “glioblastoma” was discarded for IDH-mutant tumors and should be reserved for IDH-wildtype gliomas, whereas grading from grade 2 to 4 (written in arabic numbers) was maintained [7]. Histological findings of necrosis or vascular proliferation (or both) or homozygous *CDKN2A/B* deletion allow for the diagnosis of an astrocytoma, IDH-mutant, WHO grade 4 [7]. In our PMMRDIA cohort, we could not find an association between specific histological features and clinical outcome since tumors of WHO grades II and III showed the same poor outcome as grade IV tumors. With respect to the cIMPACT-NOW guidelines, we, therefore, propose PMMRDIA as a distinct grade 4 entity, without the need of grading according to histological or molecular features.

## Electronic supplementary material

Below is the link to the electronic supplementary material.

Supplementary material 1 (pdf 427 kb)
