# Supplementary material for: Primary mismatch repair deficient IDH-mutant astrocytoma (PMMRDIA) is a distinct type with a poor prognosis
Source: Acta Neuropathol. 2020 Nov 20;141(1):85–100. doi: 10.1007/s00401-020-02243-6 (PMC7785563; doi:10.1007/s00401-020-02243-6)
Supplement: Supplementary file 1 — Supplementary material 1 (pdf 427 kb) [file 401_2020_2243_MOESM1_ESM.pdf]

Supplementary Figure 1

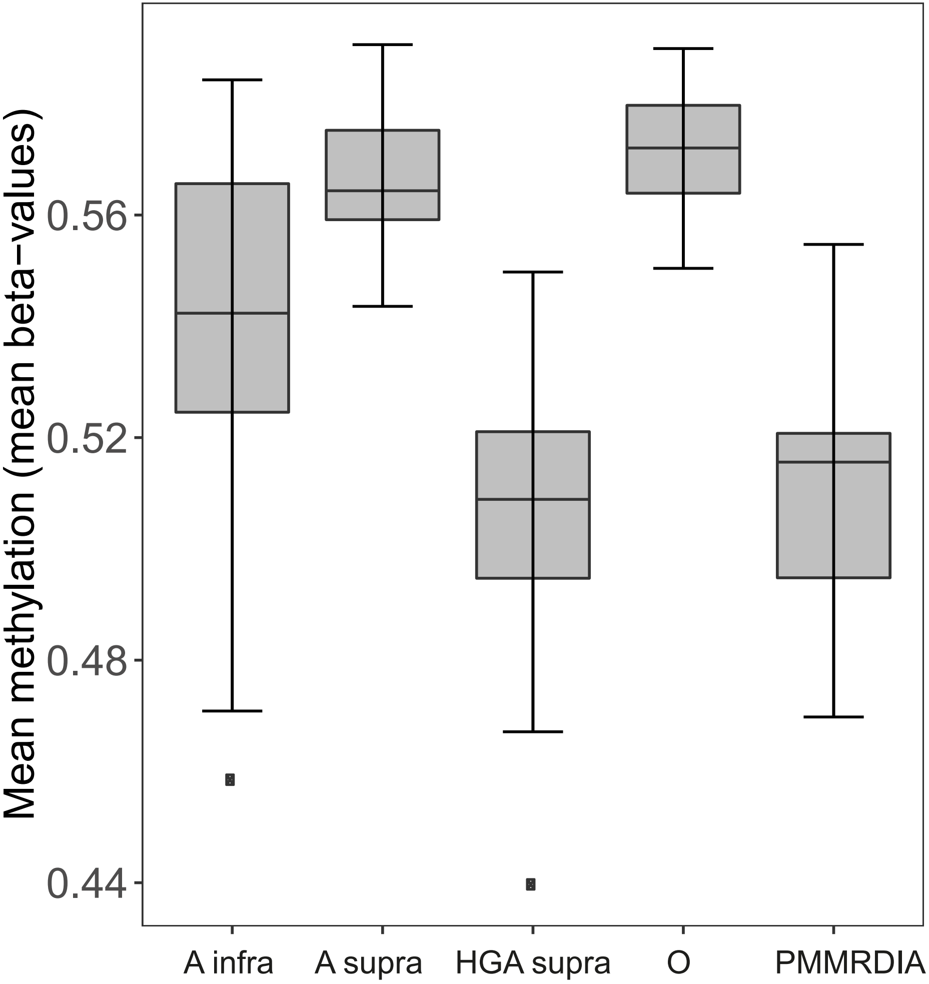

Mean methylation values among IDH-mutant gliomas. Beta-values vary from 0 (unmethylated) to 1 (fully methylated).

Supplementary Figure 2

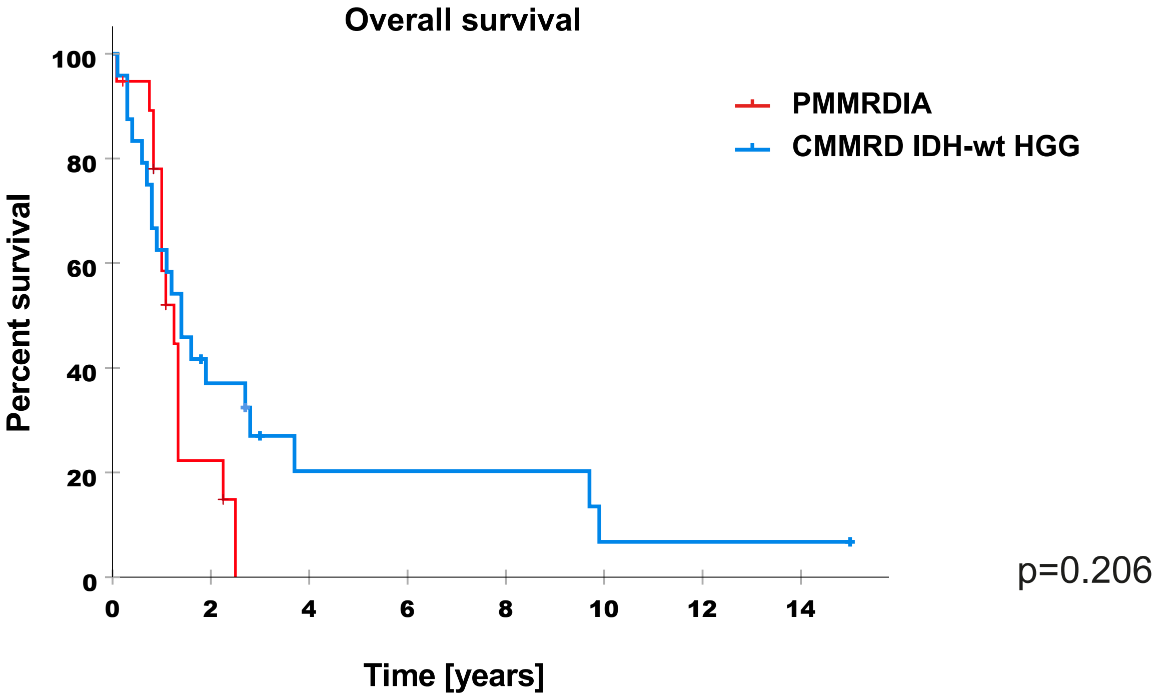

No. at risk

PMMRDIA	19	3	0	0	0	0	0	0
CMMRD IDH-wt HGG	24	8	3	3	3	1	1	1

IDH-wt glioma data was taken from
Guerrini-Rousseau et al., 2019 (PMID:32642664)

Supplementary Figure 3

a

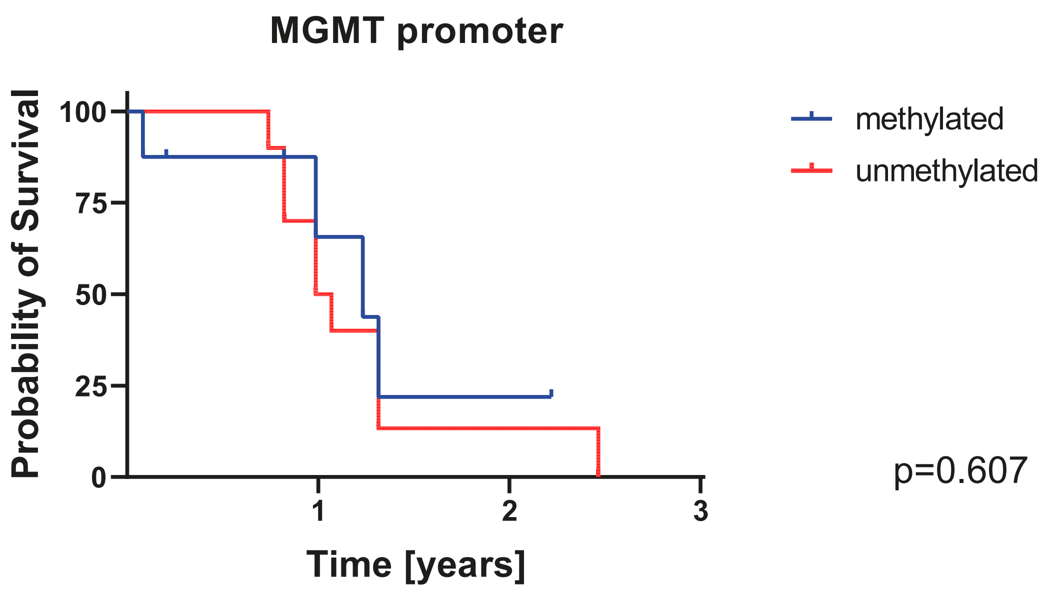

b

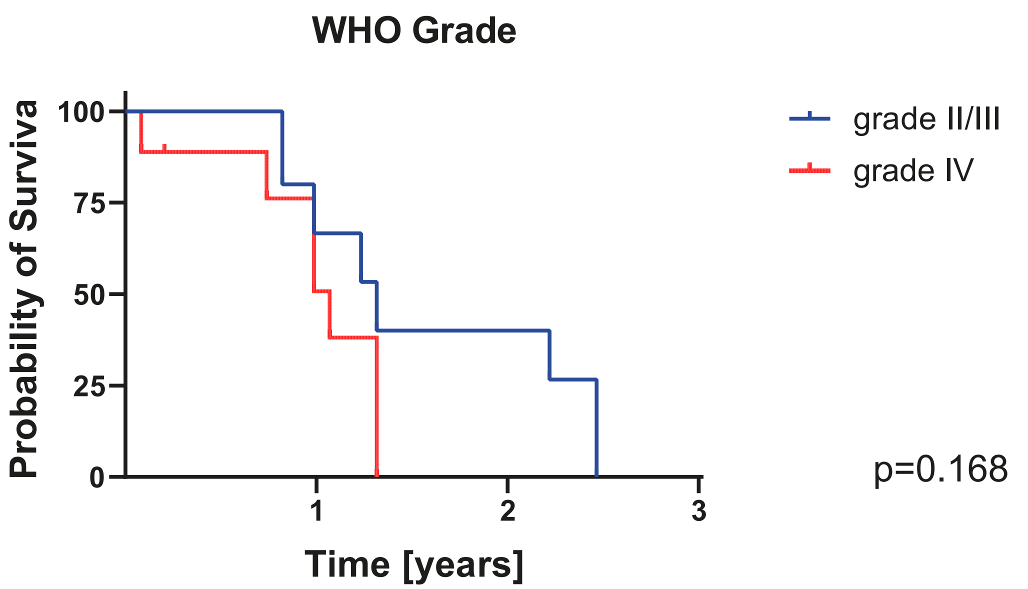

c

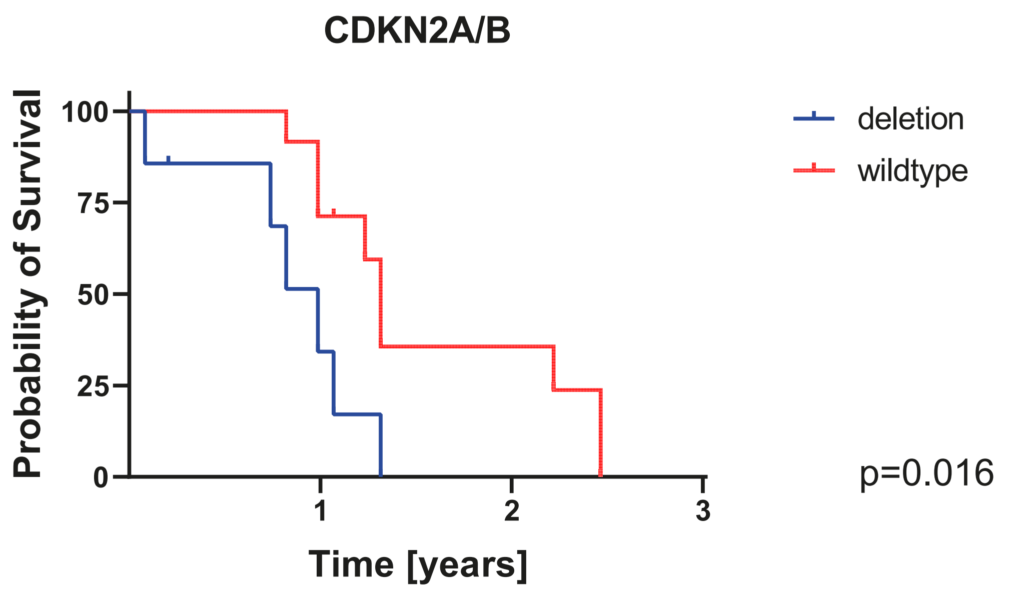

Supplementary Table 1: Treatment of PMMRDIA

No	Mut.	Syndrome	P/R	OS	PFS	Death	MGMT	Loc.	Diagn.	Grade	Treatment
1	MSH6	cMMRD	P	10	10	no	meth	H	EPN	III	
2	MLH1		P	27	3	yes	not det	H	astro	III	IR + TMZ (Stupp)
3	MSH6	cMMRD	P	1	1	yes	meth	H	GBM	IV	
4	MSH6		P	15	10	yes	meth	H	oligo	III	IR + TMZ (Stupp), CCNU, Avastin
5	MSH2		U			yes	unmeth	H	PNET	IV	
6	MSH6	Lynch	P	12	8	yes	unmeth	H	GBM	IV	IR + TMZ (Stupp), TTF, IR
7	MSH6	cMMRD	P	12	11	yes	meth	H	GBM	IV	IR, Nivolumab
8	MSH6	cMMRD	P				unmeth	H	GBM	IV	
9	MSH6		U				meth		HGG		
10			P	16	10	yes	unmeth		GBM	IV	only resection
11		cMMRD	P	16	10	yes	meth	H	GBM	IV	IR, TMZ
12	MSH6		U				unmeth		HGG		
13	MSH6	cMMRD	P	13	13	no	unmeth	H	GBM	IV	Nivolumab
14	MSH2	Lynch	P	12	6	yes	unmeth	H	astro	II	IR, TMZ, Pembrolizumab
15	MSH2	Lynch	P	6		no	unmeth	H	GBM	IV	IR, TMZ, CCNU
16	MSH6		P	16	11	yes	unmeth	H	astro	III	IR, TMZ
17			R	27	27	no	meth	H	GBM	IV	IR + TMZ (Stupp), CCNU
18			P	30	10	yes	unmeth	H	astro	III	IR + TMZ (Stupp), CCNU, Avastin
19	MSH6	cMMRD	P	10		yes	unmeth	H	astro	III	
20	MLH1	Lynch	P	10		no	meth	H	astro	III	
21	MSH6	cMMRD	P	13		yes	unmeth	PF	GBM	IV	
22	MSH6	Lynch	P	2	2	no	meth	H	GBM	IV	IR, CCNU
23			P	10		yes	unmeth	H	astro	III	IR, TMZ
24			P			yes	unmeth	H	GBM	IV	
25			P	9	9	yes	unmeth		GBM	IV	TMZ
26			U				unmeth				
27			U				meth				
28			U				unmeth				
29			U				meth				
30			U				meth				
31			U				unmeth				
32			U				unmeth				
